# Transcriptome Analysis of miRNA and mRNA in Porcine Skeletal Muscle following *Glaesserella parasuis* Challenge

**DOI:** 10.3390/genes15030359

**Published:** 2024-03-13

**Authors:** Huanhuan Zhou, Xuexue Chen, Xiangwei Deng, Xiaoyu Zhang, Xinqi Zeng, Ke Xu, Hongbo Chen

**Affiliations:** 1 Laboratory of Genetic Breeding, Reproduction and Precision Livestock Farming, School of Animal Science and Nutritional Engineering, Wuhan Polytechnic University, Wuhan 430023, China; hhzhou@whpu.edu.cn (H.Z.); chenxx_1103@163.com (X.C.); 22113033@whpu.edu.cn (K.X.); 2 Hubei Provincial Center of Technology Innovation for Domestic Animal Breeding, Wuhan Polytechnic University, Wuhan 430023, China; 3 Hubei Hongshan Laboratory, Wuhan 430070, China

**Keywords:** pig, *G. parasuis*, skeletal muscle atrophy, miRNAs, transcriptome

## Abstract

*Glaesserella parasuis* (*G. parasuis*) causes systemic infection in pigs, but its effects on skeletal muscle and underlying mechanisms are poorly understood. We investigated *G. parasuis* infection in colostrum-deprived piglets, observing decreased daily weight gain and upregulation of inflammatory factors in skeletal muscle. Muscle fiber area and diameter were significantly reduced in the treated group (*n* = 3) compared to the control group (*n* = 3), accompanied by increased expression of *FOXO1*, *FBXO32*, *TRIM63*, *CTSL*, and *BNIP3*. Based on mRNA and microRNA (miRNA) sequencing, we identified 1642 differentially expressed (DE) mRNAs and 19 known DE miRNAs in skeletal muscle tissues between the two groups. We predicted target genes with opposite expression patterns to the 19 miRNAs and found significant enrichment and activation of the FoxO signaling pathway. We found that the upregulated core effectors *FOXO1* and *FOXO4* were targeted by downregulated ssc-miR-486, ssc-miR-370, ssc-miR-615, and ssc-miR-224. Further investigation showed that their downstream upregulated genes involved in protein degradation were also targeted by the downregulated ssc-miR-370, ssc-miR-615, ssc-miR-194a-5p, and ssc-miR-194b-5p. These findings suggest that *G. parasuis* infection causes skeletal muscle atrophy in piglets through accelerated protein degradation mediated by the “miRNAs-*FOXO1/4*” axis, while further research is necessary to validate the regulatory relationships. Our results provide new insights into the understanding of systemic inflammation growth mechanisms caused by *G. parasuis* and the role of miRNAs in bacterial infection pathogenesis.

## 1. Introduction

*Glaesserella parasuis* (*G. parasuis*), formerly known as *Haemophilus parasuis*, is a Gram-negative bacterium belonging to the genus *Haemophilus* [1]. It primarily colonizes the respiratory tract of pigs and can further invade the bloodstream, leading to a systemic inflammation known as Glässer’s disease. This disease is characterized by various pathological features, including multiple serositis, arthritis, pleurisy, and meningitis [2]. The serovar diversity of *G. parasuis* is extensive, with more than 15 serovars currently recognized [3]. Among these, serovars 1, 5, 10, 12, 13, and 14 are highly virulent strains [4], with serovar 5 being the most prevalent in China based on serotyping in epidemiological studies [5]. Co-infections with other pathogens, such as porcine circovirus type 2 (PCV2) and porcine reproductive and respiratory syndrome virus (PRRSV), are common, further increasing the morbidity and mortality rates in swine [6,7,8]. In addition to the typical features of Glässer’s disease, *G. parasuis* infection in pigs is associated with clinical manifestations such as lameness, paralysis, and weight loss [9]. Skeletal muscle, as the primary power source of the motor system and a major determinant of body weight, plays a crucial role in mammalian physiology [10]. However, inflammation often leads to muscle atrophy, characterized by a rapid loss of muscle mass and myofibrillar protein [11]. Understanding the molecular mechanisms involved in skeletal muscle atrophy is crucial for devising strategies to mitigate its impact.

The regulation of skeletal muscle mass involves various molecular mechanisms, including signal transduction [12,13], transcription factors [11,12], and epigenetic modifications [14]. miRNAs are small non-coding RNAs that act as inhibitors of gene expression and play important roles in skeletal muscle development. Several miRNAs, including muscle-specific miRNAs such as those of the miR-1/miR-206 and miR-133a/miR-133b families [15,16,17,18], as well as miRNAs including miR-17 [19], miR-22 [20], miR-29 [21], miR-136 [20], miR-186 [22], miR-204 [23], and miR-222 [24], have been implicated in skeletal muscle development through their regulation of target genes. Muscle atrophy occurs when the rate of protein degradation exceeds protein synthesis, often induced by factors such as starvation and disease conditions. In many cases, miRNAs have been found to be involved in pathological and physiological stress [25,26,27,28]. Dysregulated expression of miRNAs in skeletal muscle-related diseases suggests their potential role in protecting or exacerbating skeletal muscle during disease [29,30,31,32]. Some miRNAs have been identified as non-invasive biomarkers for muscular dystrophy [33], while others have been shown to inhibit muscle atrophy and renal fibrosis or contribute to therapeutic protein restoration [34,35,36]. However, the role of miRNAs in skeletal muscle disease induced by *G. parasuis* infection remains unexplored.

In this study, we aimed to investigate the impact of *G. parasuis* infection on skeletal muscle in pigs and analyze the function of key miRNAs. We employed a combination of bioinformatics analyses and experimental verification to identify differentially expressed miRNAs and their target genes. By elucidating the potential regulatory mechanisms underlying muscle atrophy during *G. parasuis* infection, this study contributes to our understanding of bacterial infection-related diseases and provides potential targets for therapeutic intervention.

## 2. Materials and Methods

### 2.1. Animal Model Establishment and Tissue Sample Collection

The *G. parasuis*-infected colostrum-deprived (CD) piglet model was established following the methods described in previous studies [37,38]. Briefly, 15 newborn piglets that had not undergone colostrum and immunization were transported to the animal research center for artificial feeding until they reached a minimum of 21 days of age. Prior to the experiment, tests were conducted to confirm the absence of pathogens (*G. parasuis*, *Actinobacillus pleuropneumoniae*, *Streptococcus suis*, pseudorabies virus, classical swine fever virus, and PRRSV). At 30 days of age, six CD piglets were randomly selected and intratracheally injected with 2 × 10^8^ colony-forming units (CFU) of *G*. *parasuis*, specifically the SH0165 strain of serotype 5. Regular observation of clinical symptoms was conducted. Each of the piglets displayed distinct levels of clinical symptoms associated with Glasser’s disease within 48 h of infection. Among them, three piglets on the brink of death were selected for further analysis. And three unchallenged piglets were randomly selected for slaughter at 32 days of age for analysis. Swabs were taken from the lung, peritoneum, pericardium, joint fluid, and meninges to determine the extent of bacterial invasion. Longissimus dorsi muscle tissues were rapidly collected from all six piglets. One portion of the muscle tissue was cut into small cubes and fixed in 4% paraformaldehyde for histological analysis, while the other portion was cut into pieces and stored at −80 °C in a freezer after quick freezing using liquid nitrogen. All animal procedures adhered to the regulations outlined in the Hubei Provincial Regulation on Administration of Laboratory Animals (10/1/2005) and were approved by the Animal Care and Use Committee of Wuhan Polytechnical University, Hubei Province, China (WPU202108002, 1 August 2021).

### 2.2. Paraffin Section Preparation and Hematoxylin–Eosin Staining

The porcine longissimus dorsi muscle tissue block was fixed in 4% paraformaldehyde for more than 24 h. After fixation, the tissue block underwent a series of gradient dehydration steps using ethanol, starting from a low concentration and progressing to higher concentrations. Subsequently, the tissue block was then made transparent, waxed, embedded, and trimmed according to standard procedures. Cross-sections with a thickness of 3–6 µm were cut from each sample. The sections were then spread and dried, followed by dewaxing and rehydration using a series of ethanol gradients, starting from a high concentration and gradually transitioning to lower concentrations. Subsequently, the paraffin sections were stained with hematoxylin and eosin. Following staining, the sections were dehydrated, made transparent, and sealed. Hematoxylin and eosin (H&E)-stained sections were observed with a light microscope (Nexcope, Ningbo, China), and images were captured using ImageView Software (x64, 4.10.17350.20200621).

### 2.3. mRNA Library Preparation and Sequencing

Total RNA was extracted from the longissimus dorsi muscle of swine using TRIzol reagent (Invitrogen, Carlsbad, CA, USA). Each sample was replicated with three biological replicates. After agarose gel electrophoresis and NanoDrop ND-1000 quality inspection and quantification, 1–2 μg of total RNA was selected for each sample. mRNA was enriched using the NEBNext^®^ Poly (A) mRNA Magnetic Isolation Module (NEB, Ipswich, MA, USA) to remove rRNA. The RNA sequencing library was prepared using the KAPA Stranded RNA-Seq Library Prep Kit (Illumina, San Diego, CA, USA). The strand-specific library construction method based on dUTP was employed to obtain the final RNA sequencing library after PCR amplification. Subsequently, the library’s quality was assessed using the Agilent 2100 Bioanalyzer, and the library was quantified using Q-PCR. The pooled libraries from different samples were subjected to high-throughput sequencing using an Illumina NovaSeq 6000 sequencer.

### 2.4. mRNA Raw Data Processing and Expression Analysis

The raw sequencing data generated by the Illumina sequencer underwent initial evaluation using FastQC software (v0.11.9) for sequencing quality control. Then, the raw data were preprocessed to generate clean data. This preprocessing involved the pre-removed 5′ and 3′ adaptor sequences using the cutadapt tool (v3.5), as well as filtering out short fragments with a length of ≤20 bp. The reference genome (Sscrofa11 porcine reference genome) was aligned by Hisat2 software (v2.2.1). The StringTie software (v2.2.0) was employed to align the known transcriptome, and the transcript abundance was calculated. Gene-level FPKM (fragments per kilobase of gene model per million mapped fragments) values were computed using the R software (v4.1.3) package Ballgown. For gene expression analysis, a threshold of FPKM ≥ 0.5 was used to determine gene expression levels. Differentially expressed (DE) genes were identified based on the following criteria: |Fold Change| ≥ 1.50, *p*-value ≤ 0.05, q-value ≤ 1.00, and a mean FPKM value in the group ≥0.50. To visualize the results, a cluster heatmap of gene expression patterns and a volcano plot of DE genes were generated using the R software (v4.1.3) packages pheatmap and ggplot2, respectively.

### 2.5. miRNA Library Preparation and Sequencing

Three biological replicates were taken from each sample group, and tissue RNA was extracted using the TRIzol reagent (Invitrogen, USA). After agarose gel electrophoresis and NanoDrop ND-1000 quality inspection and quantification, miRNA sequencing libraries were prepared by the NEBNext^®^ Multiplex Small RNA Library Prep Set for Illumina (NEB, Ipswich, MA, USA). Following PCR amplification, PCR fragments ranging from 135 to 155 bp (corresponding to small RNAs of 14–35 nt) were selected as the final miRNA library. The quality of the miRNA libraries was assessed using the Agilent 2100 Bioanalyzer, and the mixed sample libraries were sequenced on the Illumina NextSeq 500 sequencer.

### 2.6. miRNA Raw Data Control and Expression Analysis

The raw sequencing data generated by the Illumina sequencers underwent screening using Solexa CHASTITY QC to ensure data quality. The clean reads were obtained by splitting the data with cutadapt (v3.5), with a minimum tag length of ≥17 nt. These clean reads were then aligned to the miRBase v22 database using bowtie. For quantitative analysis of the known miRNA expression, the miRDeep2 software (v1.1.1) was used to calculate the expression levels in terms of CPM (counts per million reads). The threshold for miRNA expression was set at an average CPM of each group ≥1. Differential expression analysis of miRNAs between groups was performed using the R software package edgeR. The criteria for identifying DE miRNAs were set as |Fold Change| ≥ 1.50, *p*-value ≤ 0.05, q-value ≤ 1.00, and a mean CPM in the group ≥1. To visualize the results, a cluster heatmap of miRNA expression patterns and a volcano plot of DE miRNAs were generated using the R software packages pheatmap and ggplot2, respectively.

### 2.7. miRNA Target Gene Prediction

The target genes of miRNA were predicted using two software tools, miRanda (v2.066) [39] and TargetScan (v7.2) [40]. For miRanda, the target screening parameters included “miRanda Flag” = yes, “miRanda Score” ≥ 80, and “miRanda Energy” ≤ 1.0. In TargetScan, the target filtering parameter was set as “TargetScan Flag” = yes, and “the Context plus Score” was required to be ≤−0.2. The results obtained from both methods were combined, and the genes showing an opposite expression pattern to the miRNA were considered as potential miRNA target genes for subsequent analysis. To visualize the interactions between miRNAs and their target genes, the networks were constructed using Cytoscape software (v3.9.1).

### 2.8. Functional Enrichment Analysis

To gain insights into the biological functions and pathways associated with the DE genes and miRNA target genes, Gene Ontology (GO) and Kyoto Encyclopedia of Genes and Genomes (KEGG) enrichment analyses were conducted. The OmicShare tool (https://www.omicshare.com/tools, accessed on 22 September 2023) was utilized for these analyses. For GO enrichment analysis, all differentially expressed genes were examined, and a q-value threshold of <0.05 was set to determine significantly enriched GO terms. Terms meeting this criterion were considered to be biologically relevant. Similarly, for KEGG enrichment analysis, all DE genes were investigated, and a q-value < 0.05 was used as the significance threshold to identify significantly enriched pathways. Furthermore, Gene Set Enrichment Analysis (GSEA) was performed on all expressed genes to identify enriched entries. A *p*-value < 0.05 and a q-value < 0.25 were considered significant for GSEA analysis. The OmicShare tool was employed for the above-mentioned functional enrichment analyses.

### 2.9. Quantitative Real-Time PCR

Total RNA from the longissimus dorsi muscle was extracted and reverse transcribed into cDNA using the PrimeScript RT reagent kit with gDNA Eraser (TaKaRa, Osaka, Japan) following the manufacturer’s instructions. The reference gene GAPDH was used for data normalization in mRNA analysis. For miRNA analysis, the reverse transcription process was similar to mRNA reverse transcription, but specific primers were required, and U6 was used as the internal reference gene. Q-PCR was conducted using TB Green Premix EX Taq (TaKaRa, Japan) on QuantStudio^TM^ 1 Plus real-time fluorescence quantitative PCR instrument (Thermo Fisher Scientific, Waltham, MA, USA). All samples were analyzed in triplicate. The reaction mixture for each well on the 96-well plate consisted of 5 μL of TB Green Premix EX Taq, 0.2 μL of forward primers, 0.2 μL of reverse primers, 1 μL of template cDNA, and 3.6 μL of RNA-free water. The reaction conditions followed the manufacturer’s instructions, including an initial denaturation step at 95 °C and 40 cycles of PCR amplification (95 °C for 10 s, 60 °C for 30 s). The relative expression levels of mRNAs and miRNAs in the Q-PCR data were calculated using the 2^−ΔΔCT^ method [41]. The primer sequences for inflammatory cytokines, mRNAs, and miRNAs can be found in Table S1.

### 2.10. Statistical Analyses

All Q-PCR data are presented as mean ± standard deviation (SD). Statistical significance between the control and treated groups was determined using Student’s *t*-test. A *p*-value of less than 0.05 was considered statistically significant.

## 3. Results

### 3.1. The Effect of G. parasuis Infection on Skeletal Muscle in Piglets

**Figure 1:**
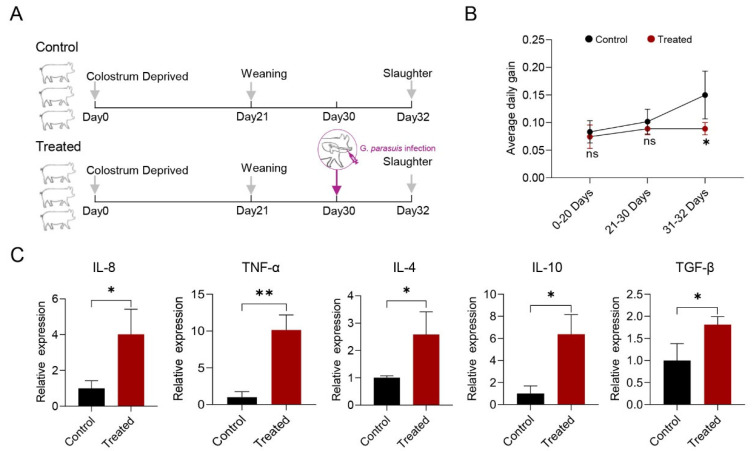
Assessment of *G. parasuis* infection in colostrum-deprived (CD) piglets. (**A**) Schematic diagram illustrating the construction of *G. parasuis*-infected CD piglet model. (**B**) Changes in average daily gain of piglets from birth to slaughter at three different stages in the treated and control groups. The stages include 0–20 days (birth to pre-weaning), 21–30 days (post-weaning to pre-infection), and 31–32 days (post-infection to slaughter). The number of piglets per group is indicated as “*n* = 3”. (**C**) Expression levels of inflammatory cytokines in skeletal muscle determined by Q-PCR. The fold change in expression is relative to the control group. * *p* < 0.05; ** *p* < 0.01.

To investigate the impact and underlying mechanism of *G. parasuis* infection on skeletal muscle, an in vivo model was established by challenging CD piglets with *G. parasuis* (Figure 1A), and the *G. parasuis* invasion detections conducted unequivocally demonstrated positive results exclusively within the treated group as our previous study described [37]. The daily weight gain of piglets was monitored following bacterial infection, indicating a substantial decline in response to the infection (Figure 1B), suggesting a potential alteration in skeletal muscle mass. To access the inflammatory response induced by *G. parasuis* in skeletal muscle, the expression patterns of inflammatory cytokines (*IL-8*, *TNF-α*, *IL-4*, *IL-10*, and *TGF-β*) were evaluated via Q-PCR in the longissimus dorsi muscle 48 h post-infection. The results demonstrated a significant upregulation of mRNA levels of these cytokines in the treated group compared to the control group (Figure 1C).

**Figure 2:**
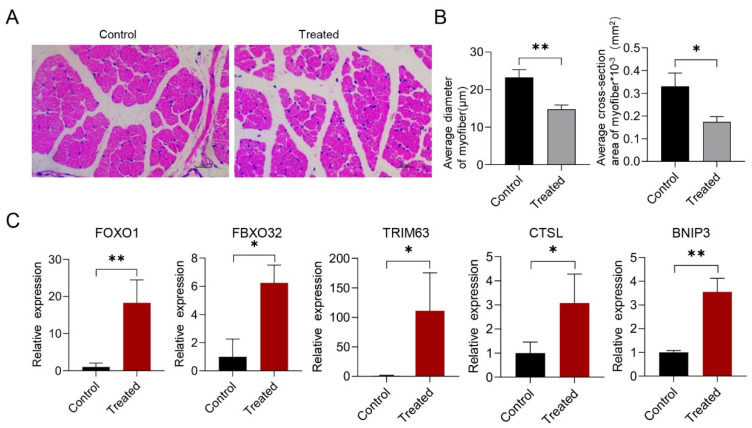
*G. parasuis* infection leading to skeletal muscle atrophy. (**A**) Representative H&E staining images of skeletal muscle isolated from control or treated piglets. Magnification: 400×; scale bars: 25 μm. (**B**) Average diameter and cross-sectional area of myofiber measured using ImageJ 1.46r software. Data are presented as mean ± SD. Statistical significance was determined using Student’s *t*-test. (**C**) Expression levels of *FOXO1*, *FBXO32*, *TRIM63*, *CTSL,* and *BNIP3* in skeletal muscle via Q-PCR. The expression fold change is relative to the control group. * *p* < 0.05; ** *p* < 0.01.

Furthermore, histological analysis was performed on the tissue, which was sectioned and stained with hematoxylin and eosin. The treated group exhibited smaller skeletal muscle fibers compared to the control group (Figure 2A). Subsequent statistical analysis confirmed a significant reduction in the average diameter and average cross-sectional area of myofibers following infection (Figure 2B). Additionally, the mRNA levels of genes associated with muscle atrophy (*FOXO1*, *FBXO32*, *TRIM63*, *CTSL*, and *BNIP3*) were significantly increased in the treated group (Figure 2C). Therefore, our findings suggest that acute *G. parasuis* infection can elicit an inflammatory response in skeletal muscle and lead to skeletal muscle atrophy characterized by smaller muscle fibers in piglets.

### 3.2. Identification of Differentially Expressed (DE) mRNAs

Six cDNA libraries were constructed using longissimus dorsi muscle tissues from the treated and control groups. The raw sequencing data of each library had a Q30 value of at least 90.23%, indicating high data quality. After data filtering, a total of 31,085,022 to 38,798,728 clean reads per library were obtained. Among these, 90.06% to 93.97% could be mapped to the reference genome (Table 1). These high-quality sequencing data were then used for further quantitative analysis of mRNA expression.

**Figure 3:**
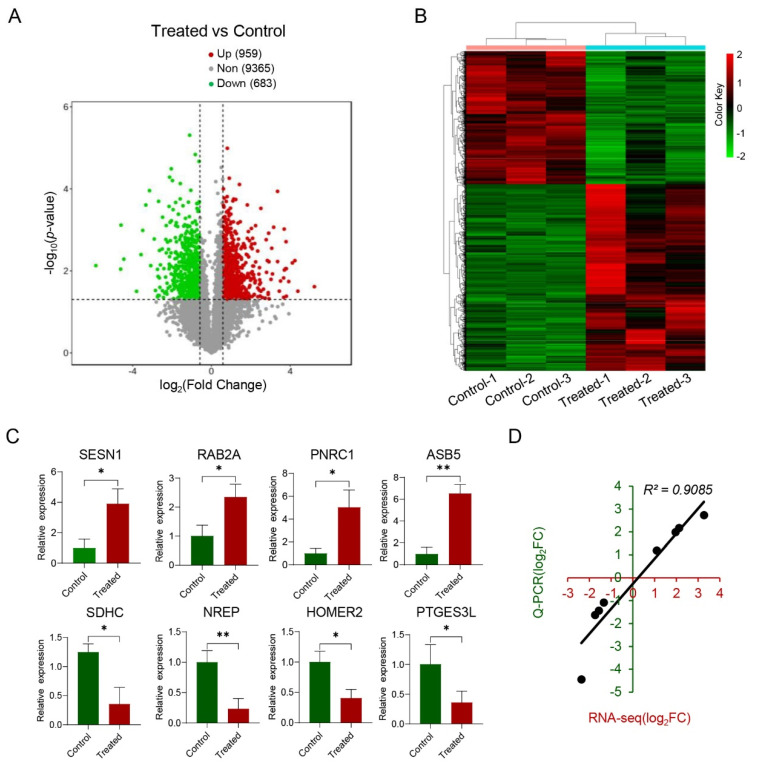
Analysis of DE mRNAs between the treated and control groups. (**A**) Volcano plot of DE mRNAs in skeletal muscle comparing the treated and control groups, determined by RNA-seq data. (**B**) Heat map of DE mRNAs generated by biclustering analysis. The values displayed represent the FPKM of the DE genes. (**C**) Verification of differentially expressed mRNAs using Q-PCR. (**D**) Pearson’s correlation analysis between Q-PCR and RNA-seq data. The x-axis and y-axis represent the log_2_(FC) data of the 8 genes between the groups, as determined by Q-PCR and RNA-seq methodologies, respectively. * *p* < 0.05; ** *p* < 0.01.

A total of 1642 DE mRNAs were identified in the treated group compared to the control group (Table S2), of which 959 were upregulated and 683 were downregulated (Figure 3A). Principal component analysis (PCA) of the DE mRNA expression patterns showed that the six samples could be clustered into two distinct groups (Figure S1A). Furthermore, biclustering analysis revealed that the DE mRNAs were not only clustered together within the same group but also exhibited roughly opposite regulatory trends between the two groups (Figure 3B). In addition, the expression patterns of four randomly selected upregulated genes (*SESN1*, *RAB2A*, *PNRC1*, and *ASB5*) and four randomly selected downregulated genes (*SDHC*, *NREP*, *HOMER2*, and *PTGES3L*) were confirmed using Q-PCR. The results indicated that all of these genes were significantly differentially expressed between the two groups (Figure 3C), and the findings were highly consistent with the corresponding RNA-seq data (*R*^2^ = 0.9085, *p* < 0.05; Figure 3D).

### 3.3. Functional Annotation of Differentially Expressed mRNAs

**Figure 4:**
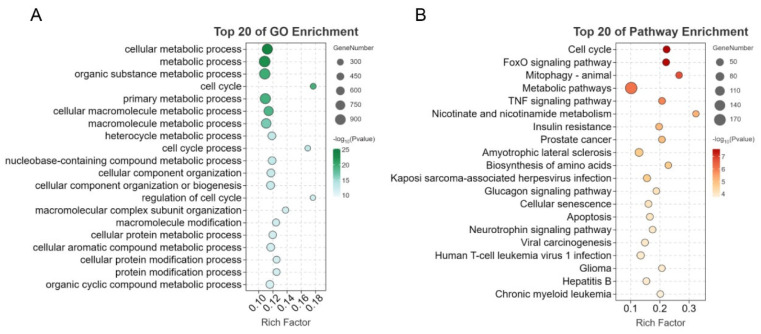
Functional annotation and enrichment analysis of DE mRNAs between the two groups. (**A**) Top 20 significant GO terms of DE mRNAs. The y-axis represents the GO enrichment in biological process, and the x-axis represents the enrichment factor. (**B**) Top 20 significantly enriched KEGG pathways of DE mRNAs. The y-axis represents the pathway, and the x-axis represents the enrichment factor.

To enhance our understanding of the DE mRNAs in *G. parasuis*-infected and normal skeletal muscles, functional enrichment analysis was conducted. GO analysis categorized the 1642 DE mRNAs into three main categories. In the biological process category, the top 20 enriched terms primarily involved metabolism-related processes. These terms included cellular metabolic process, metabolic process, organic substance metabolic process, primary metabolic process, cellular macromolecule metabolic process, macromolecule metabolic process, heterocycle metabolic process, nucleobase-containing compound metabolic process, cellular protein metabolic process, cellular aromatic compound metabolic process, and organic cyclic compound metabolic process (Figure 4A).

Regarding KEGG pathway annotation, the DE mRNAs were associated with six different classes. Among the top 20 significantly enriched pathways, four pathways (cell cycle, mitophagy-animal, cellular senescence, and apoptosis) were related to cellular processes, while the FoxO signaling pathway and TNF signaling pathway fell in environmental information processing (Figure 4B). Furthermore, three pathways (metabolic pathways, nicotinate and nicotinamide metabolism, and biosynthesis of amino acids) were associated with metabolism, and nine pathways, including amyotrophic lateral sclerosis, were related to diseases.

### 3.4. Analysis of Differentially Expressed miRNAs

For the six miRNA sequencing datasets, a total of 8,501,670 to 10,129,573 raw reads were generated per library, with a Q30 value of approximately 92% (Table 2). After removing low-quality reads, adaptors, and sequences shorter than 17 nucleotides in length, 95.70% to 98.81% of the raw reads were retained as clean reads. Among the clean reads, 78.74% to 84.05% were successfully mapped to the reference sequence in each library. These high-quality sequencing data were then subjected to quantitative analysis of miRNA expression.

**Figure 5:**
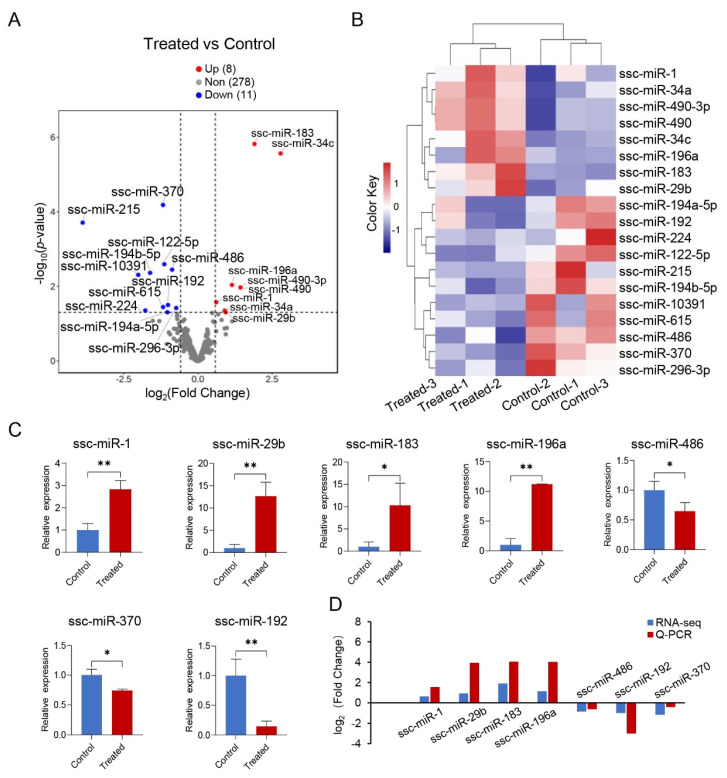
Differential miRNA expression analysis between the treated and control groups by miRNA-seq. (**A**) Volcano plot illustrating the differential expression of miRNAs in skeletal muscle between treated and control. (**B**) Biclustering analysis of the 19 differentially expressed (DE) miRNA expression profiles (CPM) in the two groups. (**C**) Expression levels of miRNAs as quantified by Q-PCR, represented as fold change relative to the control group. (**D**) Comparison of miRNA expression patterns evaluated by miRNA-seq and Q-PCR, presented as log2(FC). * *p* < 0.05; ** *p* < 0.01.

Of the known miRNAs, 19 exhibited differential expression between the two groups, with 8 miRNAs upregulated and 11 miRNAs downregulated (Figure 5A; Table S3). PCA demonstrated a distinct separation of the six samples into two clusters along the PC1 dimension (Figure S1B). Biclustering analysis revealed that the upregulated miRNAs formed one cluster, while the downregulated miRNAs formed another cluster, indicating an overall opposite expression pattern between the treated and control groups (Figure 5B). Additionally, seven randomly selected miRNAs (ssc-miR-1, ssc-miR-29b, ssc-miR-183, ssc-miR-196a, ssc-miR-486, ssc-miR-370, ssc-miR-192) from this set were subjected to Q-PCR validation, and their expression levels exhibited significant differences following *G. parasuis* infection (Figure 5C). Importantly, the expression patterns observed through Q-PCR were highly consistent with the corresponding sequencing data (Figure 5D).

### 3.5. Functional Enrichment Analysis of Predicted Targets of DE miRNAs

**Figure 6:**
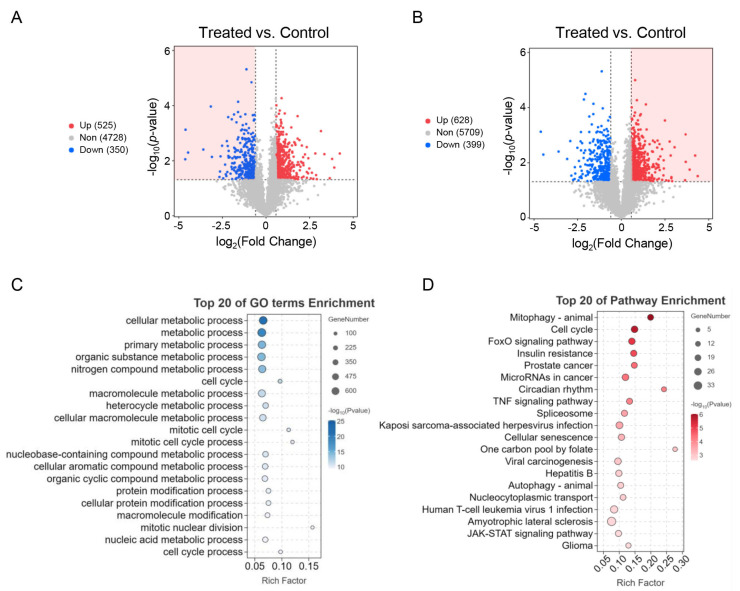
Function and pathway enrichment analysis of DE miRNA target mRNAs in skeletal muscle between the control and treated groups. (**A**) Volcano plot showing all target mRNAs of the 8 upregulated miRNAs. The red shading represents the final set of miRNA targets that exhibited an expression pattern opposite to that of the corresponding upregulated miRNAs. (**B**) All target mRNAs of the 11 downregulated miRNAs displayed by volcano plot. The red shading indicates the final set of miRNA targets of the downregulated miRNAs. (**C**) GO annotation results of DE miRNA targets. The y-axis represents the top 20 significantly enriched biological processes. (**D**) Top 20 significant pathways identified through the KEGG enrichment analysis of DE miRNA targets.

To comprehensively understand the biological functions of the 19 DE miRNAs, we utilized miRanda and TargetScan to predict a total of 85,522 potential targets, which included 13,576 unique genes (Table S4). Analysis of the targets of the eight upregulated DE miRNAs revealed 525 upregulated DE genes (Table S5), 350 downregulated DE miRNAs (Table S6), and 4728 genes that showed no significant differential expression (Table S7, Figure 6A). Similarly, the targets of the eleven downregulated DE miRNAs included 628 upregulated DE genes (Table S8), 399 downregulated DE miRNAs (Table S9), and 5709 genes that were non-differentially expressed (Table S10, Figure 6B). The final set of miRNA target genes was selected based on their expression patterns being opposite to their potentially interacting miRNAs (shaded parts of Figure 6A,B).

The 978 mRNAs targeted by the 19 DE miRNAs in skeletal muscle from the two groups were associated with multiple GO terms and pathways. Similar to the DE mRNAs, the targeted DE mRNAs were mainly enriched in biological processes related to metabolism and cell cycle (Figure 6C). KEGG pathway analysis revealed significant enrichment of the targeted DE mRNAs in pathways such as mitophagy-animal, cell cycle, FoxO signaling pathway, insulin resistance, prostate cancer, microRNAs in cancer, circadian rhythm, TNF signaling pathway, spliceosome, kaposi sarcoma-associated herpesvirus infection, cellular senescence, one carbon pool by folate, viral carcinogenesis, hepatitis B, autophagy-animal, nucleocytoplasmic transport, human T-cell leukemia virus 1 infection, amyotrophic lateral sclerosis, JAK-STAT signaling pathway, and glioma (Figure 6D).

### 3.6. The GSEA Analysis of Expression Genes

**Figure 7:**
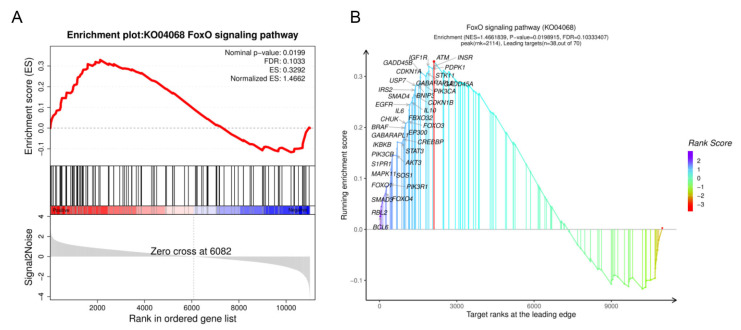
GSEA analysis of the representative significantly enriched gene set. (**A**) GSEA results of FoxO signaling pathway, comparing the treated group to the control group. (**B**) The core enrichment genes of FoxO signaling pathway identified through GSEA. The *p*-value was determined by nominal *p*-value; the q-value was determined by FDR. ES, enrichment score; NES, normalized enrichment score.

Based on the results of the KEGG and GO enrichment analyses, it was found that the FoxO signaling pathway was significantly enriched in both the DE mRNA set and the miRNA target set. This suggests that there is a strong correlation between the FoxO signaling pathway and miRNA-mediated *G. parasuis* infection in porcine skeletal muscle. Furthermore, a GSEA was conducted using all expressed genes, and it also revealed a significant enrichment of the FoxO signaling pathway. Moreover, the genes within the FoxO signaling pathway showed prominent enrichment at the top of the ranking list of all expressed genes, as depicted in Figure 7A. These findings indicate that there is a general upregulation of the gene set and activation of the FoxO signaling pathway in response to *G. parasuis* infection. In the enriched genes, 38 core enrichment genes were identified, including *FOXO1* and *FOXO4* (Figure 7B). This suggests that *FOXO1*, *FOXO4*, and other identified genes likely play key regulatory roles in the activation of the FoxO signaling pathway during *G. parasuis* infection in porcine skeletal muscle.

### 3.7. Integrated Analysis of mRNAs and miRNAs

**Figure 8:**
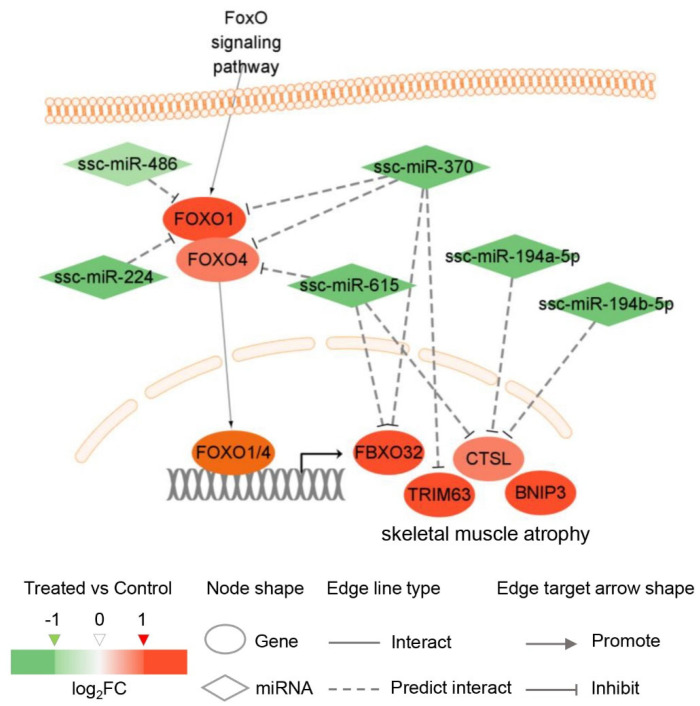
Construction of the potential miRNA-target negative correlation regulatory network involved in the FoxO signaling pathway following *G. parasuis* infection.

The constructed regulatory network in Figure 8 highlights the potential regulatory interactions between miRNAs and their target genes in the context of skeletal muscle atrophy induced by *G. parasuis* infection. In this network, miRNAs are implicated in the regulation of skeletal muscle atrophy by inhibiting the expression of *FOXO1/4* and their downstream genes. The upregulated genes *FOXO1/4*, which are core enrichment genes in the activated FoxO signaling pathway, are predicted to be inhibited by four downregulated miRNAs: ssc-miR-615, ssc-miR-486, ssc-miR-370, and ssc-miR-224. Furthermore, their downstream genes such as *FBXO32*, *TRIM63*, *CTSL*, and *BNIP3*, which have been associated with skeletal muscle atrophy in humans and mice, were found to be significantly upregulated in *G. parasuis*-infected skeletal muscle. These genes are predicted to be suppressed by four significantly downregulated miRNAs: ssc-miR-615, ssc-miR-370, ssc-miR-194a-5p, and ssc-miR-194b-5p. The downregulation of these miRNAs may contribute to the derepression of these genes, leading to their upregulation during *G. parasuis* infection. Moreover, the expression patterns of both the mRNAs and miRNAs have been experimentally validated through Q-PCR, as depicted in Figure 2 and Figure 5.

## 4. Discussion

Glässer’s disease, caused by *G. parasuis* infection, has led to huge economic losses in the pig industry. The infection triggers systemic acute inflammation characterized by multiple serositis and arthritis, as well as symptoms related to skeletal muscle function. Numerous studies have highlighted the powerful role of miRNAs in skeletal muscle size, repair, and related diseases. However, the specific effects and molecular mechanisms of *G. parasuis* infection on skeletal muscle, particularly the regulating mechanisms of miRNAs, remain unclear.

In this study, we conducted a systematic assessment of *G. parasuis* infection on skeletal muscle by analyzing daily weight gain records, detecting inflammatory factors, and examining H&E sections. We confirmed a strong inflammatory response characterized by high expression of inflammatory cytokines in skeletal muscle due to *G. parasuis* infection. This finding aligns with the well-known predilection sites of *G. parasuis* infection, such as the lung, arthrosis, pleura, peritoneum, kidney, and meninges [42,43,44]. It has been reported that *G. parasuis* breaks through the lung’s immune defense [45], allowing it to colonize various parts of the body, including skeletal muscle, through the bloodstream. In fact, *G. parasuis* has been isolated from the masseter muscle of previously specific pathogen-free (SPF) sow with acute myositis following the bacteria infection [46]. Studies have shown that *G. parasuis* can adhere to and invade different types of epithelial/endothelial cells including PUVEC [47], AOC-45 [48], PBMEC [49], PK-15 [50], NPTr, and SJPL [51], implying a capability of the bacteria to invade the bloodstream for replications and cause severe inflammation in the infected organs. Inflammation often leads to skeletal muscle atrophy [11,52], consistent with the common observation that the body tends to lose weight under pathological or physiological stress.

The FoxO signaling pathway was the most significantly enriched pathway and was found to be activated in the treated piglets. The FoxO transcription factors, including FOXO1, FOXO3, FOXO4, and FOXO6, are core effectors of this pathway and play a crucial role in cellular adaptation to various stress conditions [53]. In this study, several metabolism-related processes and pathways were significantly enriched, reflecting metabolic disorders in piglets after *G. parasuis* infection. Along with the inflammatory response in skeletal muscle, these stress conditions likely trigger the activation of the FoxO pathway and dysregulated miRNA expression [52]. Previous evidence has shown that activated FoxO signaling pathway and upregulated *FOXO1/2/3* can cause skeletal muscle atrophy [54,55]. Their target genes, including *FBXO32* (also known as *MAFbx*/*Atrogin-1*), *TRIM63* (also known as *MuRF1*), *CTSL*, and *BNIP3*, are commonly upregulated in various models of skeletal muscle atrophy [56,57], and their upregulation accelerates protein degradation [54,58]. In this study, we observed a sharp increase in the mRNA levels of *FOXO1* and *FOXO4* in skeletal muscle atrophy induced by *G. parasuis* in piglets. Correspondingly, the four target genes also showed consistent changes in mRNA expression, indicating increased protein degradation in the skeletal muscle of *G. parasuis*-infected piglets. Studies have shown that mice specifically overexpressing *FOXO1* in skeletal muscle experience decreased muscle mass and fiber size [59], while inhibition of *FOXO1* expression [60] or transcriptional activity [56,61] prevents muscle fiber atrophy and increases skeletal muscle mass. Similarly, enhanced nuclear localization and transcriptional activity of *FOXO4* induce myotube atrophy and increase the mRNA expression of *MAFbx* and *MuRF1* in C2C12 cells [62].

The results of GO and KEGG analysis of the 19 DE miRNA targets were highly similar to those of the 1642 DE mRNAs, suggesting that miRNAs play pivotal roles in muscle atrophy induced by *G. parasuis*, as observed in other types of muscle atrophy [33,34,35,36]. The upregulated *FOXO1/4* and their four downstream genes were potentially targeted by six downregulated miRNAs, including miR-486, miR-370, miR-615, miR-224, miR-194a-5p, and miR-194b-5p. Among them, *FOXO1* has been identified as a direct target of miR-486 in mice [63] and miR-370 in humans [64,65], supporting the reliability of our miRNA target predictions. In mice, miR-486 has been shown to inhibit skeletal muscle protein degradation and improve muscle mass by suppressing *FOXO1*-mediated protein degradation [63,66]. The circulating levels of miR-486 have been suggested as a biomarker for age-related sarcopenia [67], a condition characterized by the loss of muscle mass and strength or physical performance. Some studies have explored targeting miR-486 as a dietary intervention to attenuate age-associated muscle loss [68]. Importantly, *FOXO1*, *FOXO4*, *FBXO32*, and *TRIM63* were all targeted by downregulated miR-370 with absolutely opposite expression patterns in our results. Regarding miR-370, its downregulation in weaned piglets with acute inflammation induced by LPS, with the upregulation of *FOXO1*, *FOXO4*, *FBXO32*, and *TRIM63* in the gastrocnemius muscle [52]. In mice, miR-370-3p has been shown to directly inhibit the differentiation of C2C12 myoblasts [69]. These findings suggest a close relationship between miR-370 and skeletal muscle atrophy in piglets. The downregulation of miR-370 relieves the transcriptional repression of its targets, and the subsequent upregulation of *FOXO1* and *FOXO4* further enhances the transcription of *FBXO32* and *TRIM63*. Moreover, *FOXO4* and its downstream genes *FBXO32* and *CTSL* were targeted by miR-615-3p, which were downregulated during the differentiation of C2C12 cells into myotubes [70]. In addition, *FOXO1* was also targeted by miR-224, which has been shown to impair myoblast differentiation by stimulating macrophages to produce an inflammatory environment [28]. *CTSL* was targeted by miR-194a-5p and miR-194b-5p, which could promote muscle differentiation and inhibit ubiquitin ligases, ultimately leading to an increase in muscle fiber cross-sectional area [71]. The downregulation of these miRNAs in *G. parasuis*-infected piglets suggests a disruption in the regulation of *FOXO1* and *FOXO4*, leading to increased protein degradation and skeletal muscle atrophy. However, not all DE miRNAs involved in *FOXO1/4* deregulation in our study were significantly downregulated in the three infected piglets, such as miR-615, miR-194a-5p, and miR-224. A larger sample size and further experimental validation are required to confirm the regulatory relationship between these miRNAs and the target genes in the context of *G. parasuis* infection.

The intricate relationship between inflammation and muscle development has been the subject of extensive research [72,73]. Evidence from these studies consistently highlights that the loss of muscle mass is closely associated with an upsurge in genes associated with protein degradation. However, the mechanisms that are responsible for the regulation of these genes are variously described. Our results suggested that the “miRNA-FOXO1/4” axis triggered accelerated protein degradation, leading to skeletal muscle atrophy in the inflammatory piglets due to *G. parasuis* infection (Figure 8). Specifically, miR-486, miR-370, miR-615, miR-224, miR-194a-5p, and miR-194b-5p coordinately controlled the expression of *FOXO1/4* and their targets *FBXO32*, *TRIM63*, *CTSL*, and *BNIP3*. With *G. parasuis* infection, the regulatory role of the miRNAs was greatly restricted along with their reduced expression, resulting in the increased expression of their target genes. In turn, the upregulation of *FOXO1/4* further promoted the expression of the four downstream genes. These findings highlight the potential of miRNAs as therapeutic targets for mitigating skeletal muscle atrophy in pigs infected with *G. parasuis*. These findings provide new insights into the pathological mechanisms underlying skeletal muscle atrophy caused by *G. parasuis* infection in pigs. Targeting the dysregulated miRNAs and their downstream genes could potentially be explored as a therapeutic strategy to prevent or mitigate muscle atrophy associated with Glässer’s disease. A larger sample size and further research are needed to validate the regulatory relationships between miRNAs and their target genes and to investigate potential interventions for the prevention and treatment of muscle atrophy in *G. parasuis*-infected pigs.

## 5. Conclusions

This study demonstrates that *G. parasuis* infection can induce skeletal muscle atrophy in piglets. The activation of the FOXO signaling pathway, along with the upregulation of *FOXO1*, *FOXO4*, and their downstream genes (*FBXO32*, *TRIM63*, *CTSL*, and *BNIP3*), which are closely associated with skeletal muscle atrophy in *G. parasuis* infection, were firstly discovered. Furthermore, a cluster of potential miRNAs (miR-486, miR-370, miR-615, miR-224, miR-194a-5p, and miR-194b-5p) that synergistically contribute to the dysregulation of *FOXO1*, *FOXO4*, and their target genes were enriched. However, it is crucial to note that further research is required to validate these regulatory relationships. Nonetheless, these findings provide a valuable reference for further investigations into the molecular mechanisms underlying *G. parasuis*-induced skeletal muscle atrophy in pigs.

## Figures and Tables

**Table 1:** Summary of quality control and alignment of the mRNA sequencing data.

Samples	Raw Reads	Q30 (%)	Clean Reads	Clean Reads Ratio (%)	Mapped Reads Ratio (%)
Treated-1	35,530,548	91.75	34,966,632	98.41	92.42
Treated-2	39,459,754	91.13	38,664,210	97.9	92.81
Treated-3	33,483,658	90.44	32,747,122	97.80	91.50
Control-1	31,488,902	91.33	31,085,022	98.72	93.97
Control-2	40,461,752	90.23	38,798,728	95.89	90.06
Control-3	32,952,226	90.66	32,518,392	98.68	92.97

**Table 2:** Overview of the miRNA sequencing data.

Samples	Raw Reads	Q30 (%)	Clean Reads	Clean Reads Ratio (%)	Mapped Reads Ratio (%)
Treated-1	9,445,249	92.60	9,332,514	98.81	84.05
Treated-2	8,657,021	92.05	8,285,011	95.70	80.55
Treated-3	10,129,573	92.40	9,927,882	98.01	82.03
Control-1	9,506,066	92.31	9,343,438	98.29	82.98
Control-2	8,975,127	91.70	8,764,166	97.65	78.74
Control-3	8,501,670	92.40	8,153,429	95.90	81.95

## Data Availability

The mRNA and miRNA sequencing data generated in this study have been submitted to the NCBI Sequence Read Archive (SRA) database under the accession numbers (BioProject: PRJNA1065653).

## Supplementary Materials

The following supporting information can be downloaded at: https://www.mdpi.com/article/10.3390/genes15030359/s1, Figure S1: PCA of differentially expressed (DE) mRNAs and DE miRNAs. (A) Principal component analysis (PCA) plot showing the clustering pattern of DE mRNAs in the control and treated groups. (B) PCA plot illustrating the clustering pattern of DE miRNAs in the two groups. Table S1: Primer information of mRNAs and miRNAs used for Q-PCR. Table S2: DE mRNAs were identified between the treated and control groups. Table S3: DE miRNAs were identified between the treated and control groups. Table S4: DE miRNAs and their target mRNAs were identified between the two groups. Table S5: The upregulated DE miRNAs and their upregulated DE targets between the two groups. Table S6: The upregulated DE miRNAs and their downregulated DE targets between the two groups. Table S7. The upregulated DE miRNAs and their non-differentially expressed targets between the two groups. Table S8: The downregulated DE miRNAs and their upregulated DE targets between the two groups. Table S9: The downregulated DE miRNAs and their downregulated DE targets between the two groups. Table S10. The downregulated DE miRNAs and their non-differentially expressed targets between the two groups.
