# Supplementary figures and images for: Transcriptome Analysis of miRNA and mRNA in Porcine Skeletal Muscle following Glaesserella parasuis Challenge
Source: Genes (Basel). 2024 Mar 13;15(3):359. doi: 10.3390/genes15030359 (PMC10970282; doi:10.3390/genes15030359)

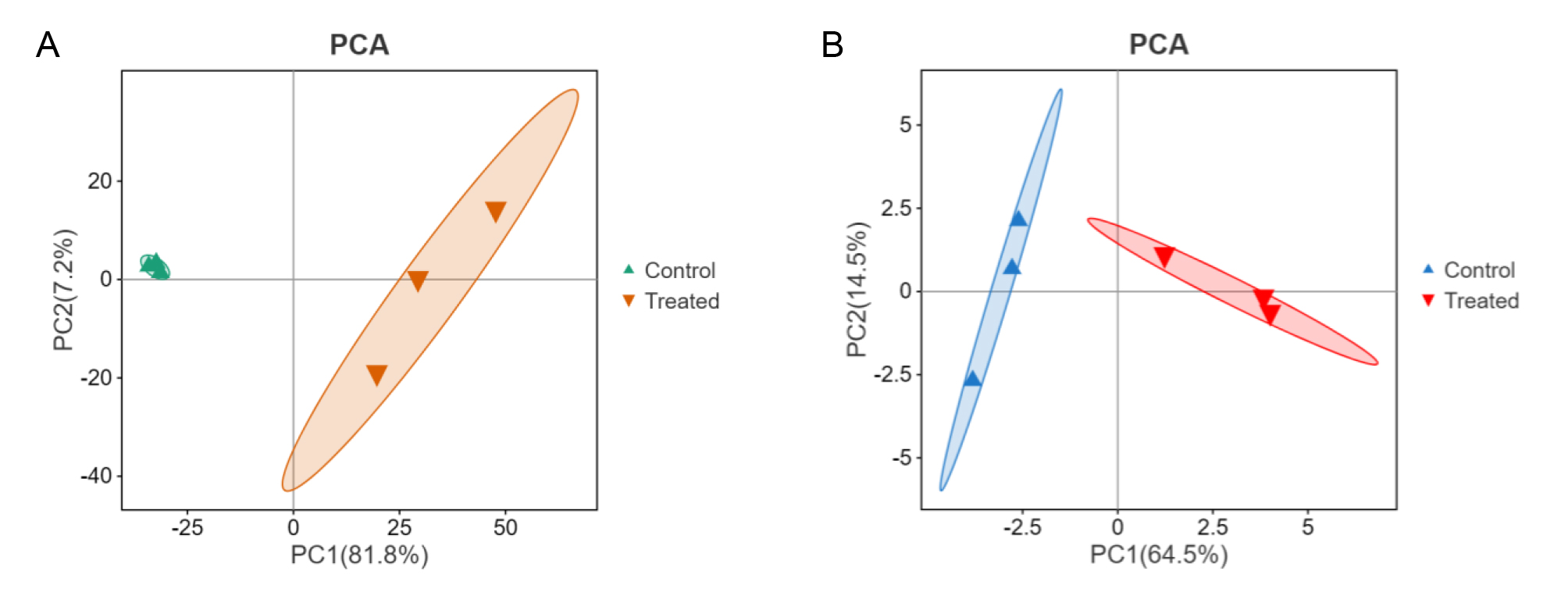

Supplement: Supplementary file 1 [file genes-15-00359-s001.zip › Figure S1.jpg]
